# Biological investigations on therapeutic effect of chitosan encapsulated nano resveratrol against gestational diabetes mellitus rats induced by streptozotocin

**DOI:** 10.1080/10717544.2020.1775722

**Published:** 2020-07-02

**Authors:** Shengye Du, Yan Lv, Na Li, Xianxia Huang, Xuemei Liu, Hui Li, Chao Wang, Yi-Fang Jia

**Affiliations:** a Department of Obstetrics, Jinan City People's Hospital, Jinan, PR China; b Gynecological Reproductive Clinic, Jinan City People's Hospital, Jinan, PR China; c Department of Gynecology and Traditional Chinese Medicine, Jinan City People's Hospital, Jinan, PR China; d Center of Prenatal Diagnosis, Shandong Provincial Hospital Affiliated to Shandong First Medical University, Jinan, PR China

**Keywords:** Gestational diabetes mellitus, chitosan, streptozotocin, resveratrol, endoplasmic reticulum stress

## Abstract

The chitosan encapsulation with bioactive compounds (resveratrol) is a significant method that can be used to raise the stability and effectiveness of substances in gestational diabetes management. In this study, the resveratrol–zinc oxide complex is encapsulated with chitosan (CS–ZnO–RS). The synthesized CS–ZnO–RS could be used to deliver the resveratrol with minimized side effects and also improved bioavailability. CS–ZnO–RS were characterized by various techniques such as particle size analyzer, DSC, FT-IR, TEM, SEM, and AFM. The electron microscopic and particle analyzer confirmed that the synthesized CS–ZnO–RS were monodispersed, spherical and its average size was 38 nm. The drug-releasing profile showed that 95% of RS is released from CS–ZnO–RS within 24 h. *In vitro* studies confirmed that α-glucosidase and α-amylase inhibitory activities were closely related to the concentration of CS–ZnO–RS. The highest inhibition of α-glucosidase (77.32%) and α-amylase (78.4%) was observed at 500 μg/mL. Furthermore, the treatment of CS–ZnO–RS significantly decreased the blood glucose levels in gestational diabetes mellitus induced rats and maintained the lipid content toward the normal rats. In addition, the CS–ZnO–RS reduced the level of inflammation factors (IL-6 and MCP-1) and endoplasmic reticulum stress (GRP78, p-IRE1α, p-eIF2α, and p-PERK).

## Graphical Abstract

**Figure:**
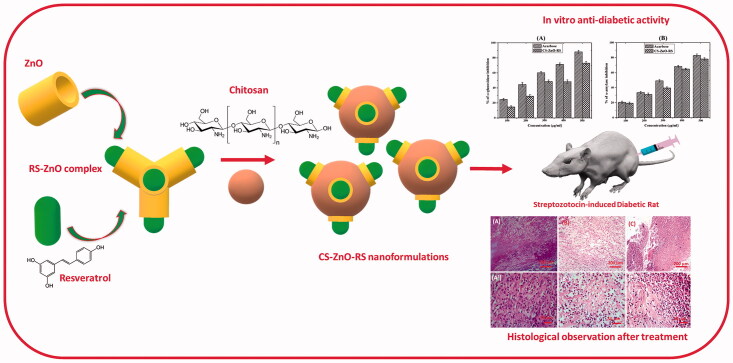

## 1. Introduction

Diabetes mellitus (DM) is a kind of universal health problem in the 21st century. It is estimated that 20.9 million live births were affected, an astounding one in seven childbirths (IDF, 2015). The higher level of blood glucose during pregnancy which can be classified into two types namely gestational diabetes mellitus (GDM) and pre-gestational diabetes mellitus (PGDM). GDM is a complex disease that is characterized by increasing the blood glucose level during the gestation period (Ristagno et al., 2012). In the past few decades, the number of people affected by GDM problem has been increased. Almost 7% of all pregnant women are complicated and 3–7% of women deteriorate with long-term diabetes after pregnancy. Furthermore, GDM causes abnormal fetal development (Gardosi & Francis, 2009). Therefore, the prevention of GDM could help enlighten the health of the people. At present, the available treatments are not suitable for the prevention and managing of diabetes.

A comprehensive and elaborated study of GDM could be a necessity for the event of protective and active methods. The findings of metabolic changes are auspicious in the examination of etiological pathways. There are numerous biomarkers reported as pointers for GDM such as plasma glucose and glycated hemoglobin A1c (HbA1c), triglycerides (TGs), high-density lipoprotein (HDL), inflammatory markers, and enzymic and non-enzymic antioxidants. Metabolites are intermediate products of pathways which reproduce the various physiological dysfunctions.

The presence of secondary metabolites in natural foods clarifies the possibility to prevent and reduce the risk of several diseases. The existence of alkaloids, polyphenols, flavonoids, sterols, pigments, and fibers involves in the major role of disease prevention (Greca & Zarrelli, 2012; Vasto et al., 2014). Especially, the food rich in secondary metabolites enhances the free radical scavenging, anti-inflammatory and anti-cholesterol activity which also promotes insulin sensitivity and decreased resistance (Georgoulis et al., 2014).

Resveratrol (3, 5, 4-trihydroxystilbene) is a polyphenolic compound synthesized by more than 70 kinds of plants and several dietary substances (Roemer & Mahyar-Roemer, 2002; Baur et al., 2006). It has been reported that it possesses several beneficial activities such as prevention of insulin resistance (IR), increases the mitochondrial contents of various tissues as well as upgrades the normal performance in obese mice (Baur et al., 2006; Pearson et al., 2008; Baur, 2010). Recent studies proved that the administration of resveratrol improved IR as well as reduced the blood glucose, TGs, cytokines, lipid and blood pressure (Olholm et al., 2010; Timmers et al., 2011). Still, there are no detailed molecular pathway studies on resveratrol against GDM. In this study, a different approach was carried out to enhance the bioavailability of resveratrol by developing a resveratrol–metal complex and encapsulated with chitosan nanoparticles (CS-NPs). On the other hand, zinc metal involved in the major role of synthesis and regulated the insulin secretion by controlling the blood glucose level (Prasad, 2013). Moreover, a zinc deficient person has reduced insulin sensitivity and increased level of glucose (Kim & Lee, 2012). Therefore, the supplementation of zinc elements applied in this nanocomposite could resolve the zinc deficiencies.

Here, the mechanism and action of chitosan encapsulated nano resveratrol is against the GDM. The prepared resveratrol-ZnO-chitosan (CS–ZnO–RS) was characterized by various techniques including particle size analyzer, DSC, FT-IR, TEM, SEM, and AFM. Furthermore, the anti-gestational diabetes mellitus activity was investigated by *in vitro* and *in vivo* studies.

## 2. Materials and methods

### 2.1. Chemical and reagents

Streptozotocin, chitosan, and resveratrol were obtained from Sigma-Aldrich Co. (St. Louis, MO). The chemical and reagents used for antioxidant assays were purchased from Himedia Laboratories (West Chester, PA). The one-touch glucometer (ACCU-CHEK Sensor, Mannheim, Germany) was used to monitor the blood glucose level.

### 2.2. Synthesis of zinc oxide nanoparticles

The zinc oxide nanoparticles (ZnO-NPs) were synthesized by following the procedure mentioned by Dhanavel et al. (2014). Briefly, 0.05 M NaOH solution was added into the zinc acetate solution (0.01 M) under stirring condition. The precipitate was washed with double distilled water and dried at room temperature. The collected powder sample was calcined at 430 °C for 30 min and used for further studies.

### 2.3. Chitosan encapsulated nano resveratrol and zinc oxide nanoparticles

In this study, the nanoparticles were successfully encapsulated with chitosan molecules through ion gelation method (Calvo et al., 1997). Briefly, the ZnO-NPs were dissolved in 1% of the acetic acid solution and it was added into the CS-NPs under stirring condition. The resveratrol was dissolved separately in ethanol and was added to the CS-NPs solution placed on a magnetic stirrer at room temperature to obtain the CS–ZnO–RS solution. TPP solution (0.02%) was added to CS–ZnO–RS solution and kept on a magnetic stirrer at 25 °C for 90 min. Then, the solution was centrifuged at 1200 rpm and the collected supernatant was concentrated by vacuum evaporator. The obtained CS–ZnO–RS was dissolved in a minimum volume of absolute ethanol and it was allowed to sonication for 5 min (60 Hz and 30 s cycle). Finally, the CS–ZnO–RS solution was filtered through the Whatman No. 1 filter paper and its volume was concentrated by vacuum evaporator.

### 2.4. Characterization studies

The size of CS–ZnO–RS was determined by particle size analyzer (Malvern HPPS 5001 particle size analyzer, Malvern, UK). About 5 mL of aquadest was mixed with a few drops of CS–ZnO–RS and subjected to a size analyzer. The physical condition of CS–ZnO–RS was examined by differential scanning calorimetry (DSC) analysis. The functional groups were detected by Fourier transform infrared spectroscopy (FT-IR) analysis (PerkinElmer, spectrum two, Waltham, MA). The CS–ZnO–RS was mixed with potassium bromide (KBr) pellets and the spectrum was measured between the range of 4000 and 400 cm^−1^. The size, shape, and surface morphology were studied by the assistance of transmission electron microscope (JEOL JEM-1200EX microscope, Tokyo, Japan), scanning electron microscope (JEOL SEM, Tokyo, Japan) and atomic force microscope (Bruker Multimode 8, Billerica, MA) analysis.

### 2.5. *In vitro* analysis

#### 2.5.1. α-Glucosidase activity

The α-glucosidase activity was determined by the method of Ademiluyi & Oboh (2013). Briefly, the various concentrations of CS–ZnO–RS (10–50 μg/mL) were treated with 1 U mL^−1^ of α-glucosidase solution at 37 °C for 15 min. Then, 250 μL of 4-nitrophenyl α-d-glucopyranoside (pNPG) was added and incubated again for 20 min. In this analysis, acarbose was used as a positive control. The absorbance of the solution was recorded at 405 nm.

#### 2.5.2. α-Amylase activity

The α-amylase activity was scrutinized by Shai et al. (2010) with minor changes. Different concentration of CS–ZnO–RS (25, 50, 100, 150, and 200 μg/mL) was mixed with porcine pancreatic amylase (2 U mL^−1^) solution and incubated at 37 °C for 20 min. One percent of starch was added to this mixture and extended the incubation time for 1 h. Afterwards, the reaction mixture was treated with 500 μL of DNS (3,5-dinitrosalicylate) and the absorbance was read at 540 nm.

### 2.6. *In vivo* antidiabetic activity

#### 2.6.1. Animal maintenance

The male healthy Wistar albino rats (180–200 g) were purchased from the Institutional Animal Center, Jinan People's Hospital, China. The animals were maintained at 23 ± 1 °C in a controlled environment with 12 h light and 12 h dark cycle. The GDM was induced by the exposure of streptozotocin (i.p. 45 mg/kg bw). All the experiments were carried out by specific guidelines prescribed by the Animal Ethical Committee, Jinan People's Hospital, China. Over the three days of induction, the rat with ≥250 mg/dL of blood glucose was selected for further study.

#### 2.6.2. Toxicity analysis

For the toxicity analysis, the rats were classified into two groups namely, group I: control and group II: experimental groups. The experimental group of rats was orally administrated with CS–ZnO–RS (200 mg/kg bw) for 28 days. The observed signs of toxicity were recorded.

#### 2.6.3. Drug dosage and duration of administration

According to the strategies, the dosage for toxicity examination in rodents should be higher acceptable concentration. Therefore, a maximum concentration of CS–ZnO–RS (200 mg/kg body weight) was selected for this study. All the animals were treated with 200 mg/kg body weight of CS–ZnO–RS for 28 days. The control groups of animals received normal physiological saline. At the end of the experiment, the rats were anesthetized by diethyl ether and the collected blood sample was used for hematological analysis.

#### 2.6.4. Pharmacokinetics (PK) studies

The healthy rats (150–200 g) were randomly classified into three groups. Each group contains six rats. The normal rats were served as control (group I). The rats were injected by streptozotocin (i.p.; 45 mg/kg bw) which is considered as GDM challenged group (group II). In group III, the GDM challenged rats were orally treated with CS–ZnO–RS (100 mg/kg bw). At the end of the experiment, the control and experimental groups of rats were anesthetized using diethyl ether and the blood sample was collected using cardiac puncture. The serum was separated by centrifuging the sample at 4000 rpm for 5 min. Simultaneously, liver tissue was removed immediately and subjected to histopathological studies.

#### 2.6.5. Measurement of body weight, serum glucose, and insulin

The body weight was calculated using a top loading balance. The serum glucose level was monitored using a glucometer. Further, the serum insulin level was measured by the Ultrasensitive Mouse Insulin ELISA kit (ALPCO, Salem, NH). The homeostasis model assessment for insulin resistance (HOMA-IR) was determined by previous studies (McKeown et al., 2004; Fraulob et al., 2010; Ables et al., 2012). The formula for HOMA-β (HOMA for β-cell function) examination was reported by Matthews et al. (1985) and Singh & Saxena (2010).

#### 2.6.6. Exposure of lipid profile

The total cholesterol (TC), TG, HDL, and low-density lipoprotein (LDL) contents were determined by commercial kits obtained from Cayman Chemical (Ann Arbor, MI). The atherogenic index (AI) was calculated by the following formula.

$$\mathrm{AI}=\frac{\text{total cholesterol}-\text{HDL cholesterol}}{\text{HDL cholesterol}}$$

#### 2.6.7. Antioxidant assays

The antioxidant assays such as superoxide dismutase (SOD), catalase (CAT), glutathione peroxidase (GPx), and glutathione (GSH) were determined by commercial analytical kits procured from Abcam (Shanghai, China).

#### 2.6.8. Real-time PCR (RT-PCR) analysis

Briefly, the total RNA was extracted from the placenta of control and experimental group of rats by the assistance of Trizol reagents (Thermo Fisher, Waltham, MA). Then, RNA was reverse-transcribed by Super Script^®^ III First-Strand Synthesis System (Thermo Fisher, Waltham, MA). The RT-PCR experiment was performed by the instruction mentioned in the QuantiTect SYBR Green PCR Kit (Qiagen, Germantown, MD). The details of the primers were as follows.

- IL-6: 5′-CCTCTGGTCTTCTGGAGTACC-3′, 5′-ACTCCTTCTGTGACTC CAGC-3′
- MCP-1: 5′-GCTCAGCCAGATGCAGTTAA-3′, 5′-TCTTGAGCTTGGTG ACAAA.

#### 2.6.9. Western blot analysis

The total protein was extracted from the placenta by ReadyPrep™ Protein Extraction Kit (Bio-Rad, Hercules, CA) and the concentration was determined by Bio-Rad Protein Assay (Bio-Rad, Hercules, CA). The protein was separated by SGS-PAGE and it was transferred to the PVDF membrane. Afterwards, the membrane was incubated with primary antibody (anti-GRP78, (78 kDa glucose-regulated protein 78), anti-IRE-1α (inositol-requiring enzyme-1α), anti-p-IRE1α, anti-eIF2α (eukaryotic initiation factor alpha 2), anti-p-eIF2α, and anti-β-actin) for overnight followed by HRP-conjugated secondary antibody. The immunoreactive proteins were detected by Clarity™ Western ECL Blotting Substrates (Bio-Rad, Hercules, CA) and the density was measured by GS-900™ Calibrated Densitometer (Bio-Rad, Hercules, CA).

#### 2.6.10. Histopathological studies

The liver tissue from the control and experimental group of rats was dissected out and immediately washed with normal saline and stored at neutral buffered formalin. After, the tissue was fixed in 10% of formalin using paraffin techniques. A 5 µm size of tissue section was prepared and stained with hematoxylin and eosin. The histopathological changes were observed using a bright-field microscope (Carl Zeiss Axioscop microscope, Oberkochen, Germany).

### 2.7. Statistical analysis

All the experimental data were displayed as mean ± SD. The statistical variance was studied by a one-way or two-way ANOVA using the Kruskal–Wallis test. *p* Value ˂ .05 is considered statistically significant.

## 3. Results and discussion

Gestational diabetes mellitus is considered as an initial stage of type 2 diabetes. Insulin resistance is primary pathogenesis of GDM and type 2 diabetes. There are several groups of researchers who have described the potential effect of resveratrol against diabetes challenged animals (Al-Hussaini & Kilarkaje, 2018; Gocmez et al., 2019; Xu et al., 2019). However, resveratrol with metal nanocomposites studies is very scanty. In the combined treatment, zinc involved a crucial role in pancreatic islets that increase the zinc ion contents (Maret, 2017). In this study, the effect of CS–ZnO–RS was inspected in GDM challenged rats using various biochemical parameters and antioxidant enzymes.

### 3.1. Characterization of CS–ZnO–RS

**Figure 1.**
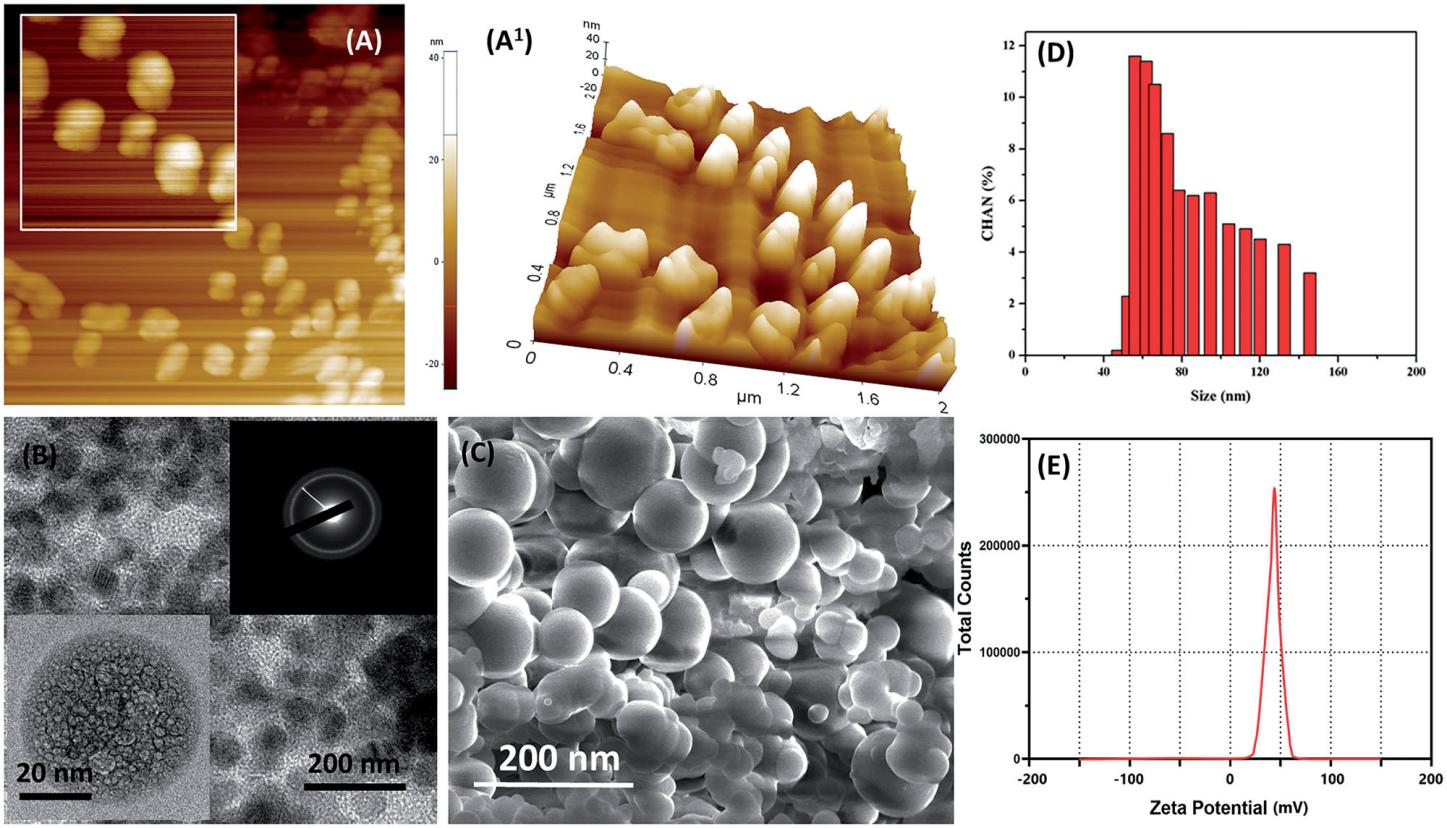
Microscopic characterization of CS–ZnO–RS; (A, A_1_) AFM, (B) TEM (inserted SAED), (C) SEM, (D) particle size analysis, and (E) zeta potential results of prepared CS–ZnO–RS nanoformulation.

**Figure 2.**
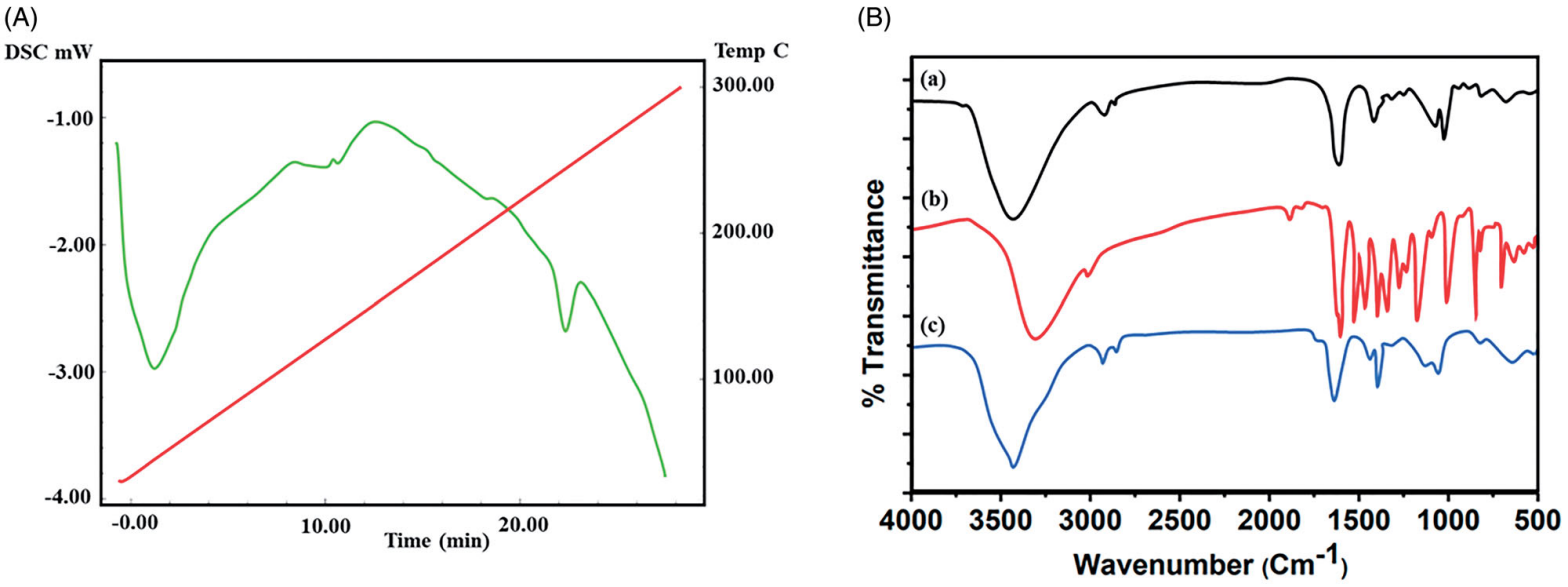
(A) DSC and (B) FTIR spectrum (a, CS; b, RS; c, CS–ZnO–RS) of CS–ZnO–RS.

The synthesized nanoparticles were encapsulated with chitosan molecules through ion gelation method (Calvo et al., 1997). The chitosan molecules own positive electric charges due to the existence of the amine group (NH_2_) which will be protonated as NH_3_^+^. Also, the presence of amine and hydroxyl groups provides the ability to the formation of zinc metal complexes. The morphology characters such as size, shape, and surface morphology were examined by electron microscopic techniques. Mostly, the particles are monodispersed, spherical, and exist without any aggregation (Figure 1(A–C)). CS–ZnO–RS showed an average particle size at 38 nm (Figure 1(D)). The zeta potential of CS–ZnO–RS was 39.1 mV, which is displayed in Figure 1(E). The positive charges of CS–ZnO–RS cooperated that the amino groups of chitosan occurred in the surface of nanocomposite and greater than 25.0 mV which revealed that the nanoparticle suspensions were stable and not easy to aggregate (Jeong et al., 2016). The result of DSC is presented in Figure 2(A). Initially, the CS–ZnO–RS was decomposed with temperature. While increasing the temperature (150 °C), it was enhanced and completed at ∼257 °C. Furthermore, the DSC curves show a corresponding peak for ZnO at 257 °C.

IR spectrum is displayed in Figure 2(B). The CS–ZnO–RS exhibited the absorbance peaks at 3421 and 2992 cm^−1^ which correspond to the existence of aromatic C–H stretching of nano-resveratrol (Jeong et al., 2016). The presence of major peaks at 1630 and 1615 cm^−1^ indicates the *v*(C=C) and *v*(C=O) of nano-resveratrol (Cheng et al., 2018). The disappearance of peaks at 1650–1800 cm^−1^ represents the resveratrol which mainly exists in enol form rather than keto form. The peak is recorded at 1405 cm^−1^ due to the aromatic vibrations *v*(C=C) of nano-resveratrol (Jeong et al., 2016). The similar spectrum values were seen in CS–ZnO–RS. The two peaks of nano-resveratrol (1128 and 1047 cm^−1^) continued in CS–ZnO–RS spectrum which is due to the *δ* CH3 and *v* (O–CH3).

### 3.2. Determination of drug-releasing

**Figure 3.**
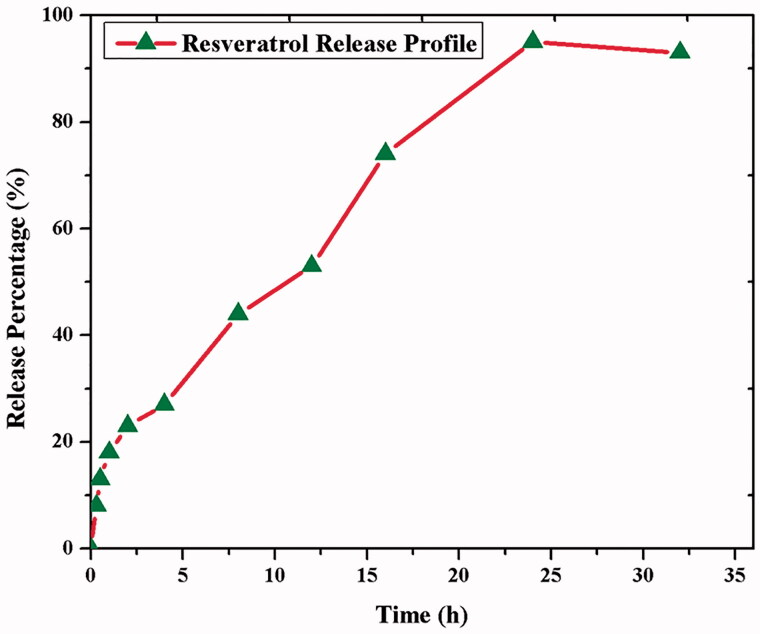
*In vitro* cumulative release of resveratrol CS–ZnO–RS nanoparticles.

The resveratrol releasing from CS–ZnO–RS was determined by PBS (pH 7.4) at room temperature. The release pattern showed the constant release of RS from CS–ZnO–RS, wherein more than 95% was released within 24 h (Figure 3). The 44% of RS releasing activity was seen in 8 h, which may be the reason for the hydrophilicity of RS. This constant release of RS from the CS–ZnO–RS offers the advantage of once-daily dosing and avoids the multiple dosing.

### 3.3. *In vitro* analysis

**Figure 4.**
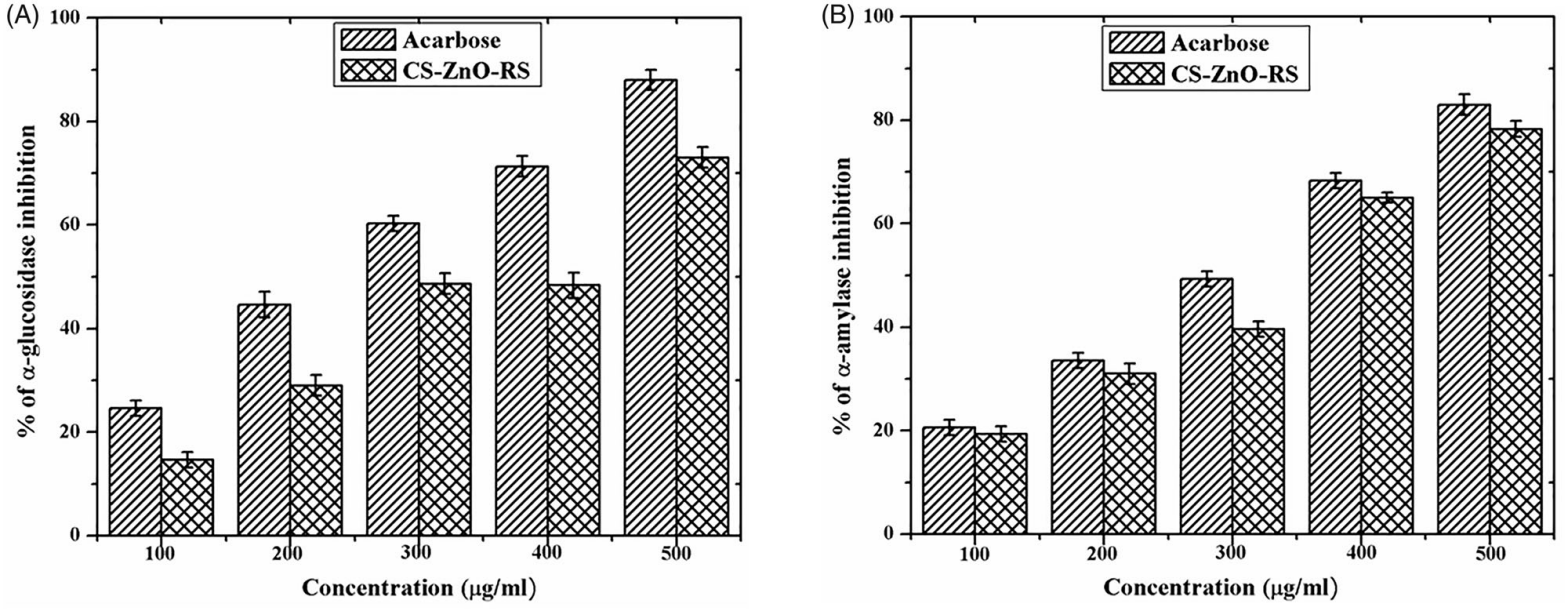
*In vitro* examination of antidiabetic activity using α-glucosidase and α-amylase.

The antidiabetic activity was determined by α-glucosidase and α-amylase inhibitory activities. The α-glucosidase and α-amylase mainly contribute carbohydrate digestion through hydrolyzing. The better way to control type 2 diabetes is an interruption of carbohydrate digestion through inhibition of α-glucosidase and α-amylase resulting in decrease of the glucose absorption (Ali et al., 2006; Loizzo et al., 2008). The inhibitory activities are displayed in Figure 4(A,B). The obtained activities were closely related to the concentration of CS–ZnO–RS. Among that, the highest activity (73%) was recorded at 500 μg/mL. The activity of CS–ZnO–RS was compared to the standard antidiabetic drug (acarbose), which exhibited the maximum α-glucosidase inhibitory activity (88%). Similarly, the α-amylase activity was tested using various concentrations of CS–ZnO–RS. CS–ZnO–RS and acarbose showed the highest activity at 78.3% and 83%, respectively.

### 3.4. *In vivo* antidiabetic activity

#### 3.4.1. Toxicity and dosage analysis

**Figure 5.**
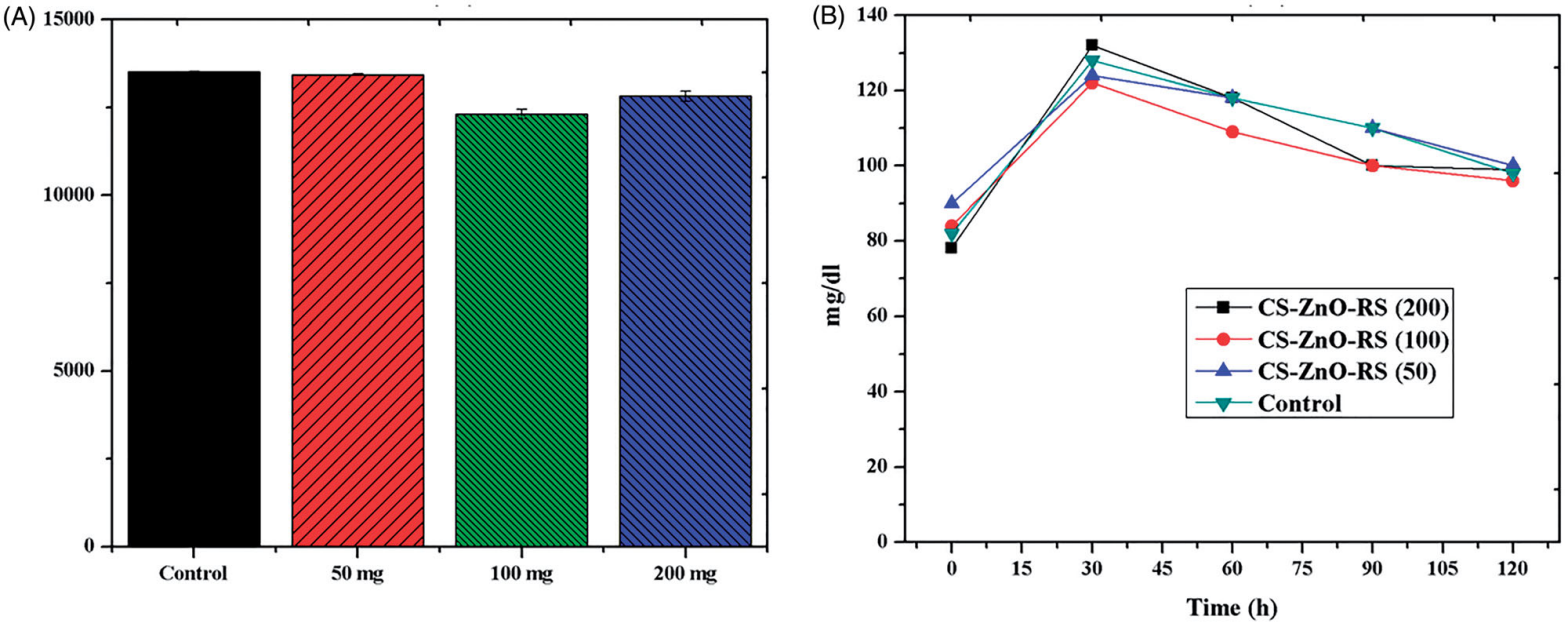
Fixation of optimum dosage level in Wister albino rats.

The toxicity effect of CS–ZnO–RS was examined by hematological parameters and the results are presented in Table 1. The control groups of animals showed RBC, HB, HCT, MCV, MCH, and MCHC to be 7.3 ± 0.3 × 10^12^/L, 12.1 ± 0.6 g/dL, 37.8 ± 0.3%, 54.2 ± 0.2 fL, 19.5 ± 0.4 pg, and 34.6 ± 0.2 g/dL. However, the rats were treated with CS–ZnO–RS which showed the parameters to be 7.4 ± 0.5 × 10^12^/L, 10.8 ± 0.3 g/dL, 38.2 ± 0.4%, 54.6 ± 0.3 fL, 18.1 ± 0.7 pg, and 32.9 ± 0.8 g/dL, respectively. While comparing the two results, there are no significant results observed. The parameters of CS–ZnO–RS treated groups were similar to the control. These findings demonstrated that the CS–ZnO–RS did not show any toxic effects in the rats. These results were mostly agreed with previous studies (Chauhan et al., 2019). On the other hand, the optimum dosage was determined by the dose fixation study. The rats were administrated with different concentrations of CS–ZnO–RS (50, 100, and 200 mg/kg bw). Over the experiment period, the maximum blood glucose reduction was obtained at 100 mg/kg bw of CS–ZnO–RS (Figure 5). Therefore, this concentration was taken for further studies.

**Table 1.** Toxicity analysis of CS–ZnO–RS.

	Variations after treatment	
Hematological parameter	Control	CS–ZnO–RS
Red blood cell count (×10^12^/L)	7.3 ± 0.3	7.4 ± 0.5
Hemoglobin (g/dL)	12.1 ± 0.6	10.8 ± 0.3
Hematocrit (%)	37.8 ± 0.3	38.2 ± 0.4
Mean cell volume (fL)	54.2 ± 0.2	54.6 ± 0.3
Mean cell hemoglobin (pg)	19.5 ± 0.4	18.1 ± 0.7
Mean cell hemoglobin concentration (g/dL)	34.6 ± 0.2	32.9 ± 0.8

Data are expressed as mean ± standard deviation (SD).

### 3.5. Pharmacokinetics studies

#### 3.5.1. Measurement of body weight, serum glucose, and insulin

**Figure 6.**
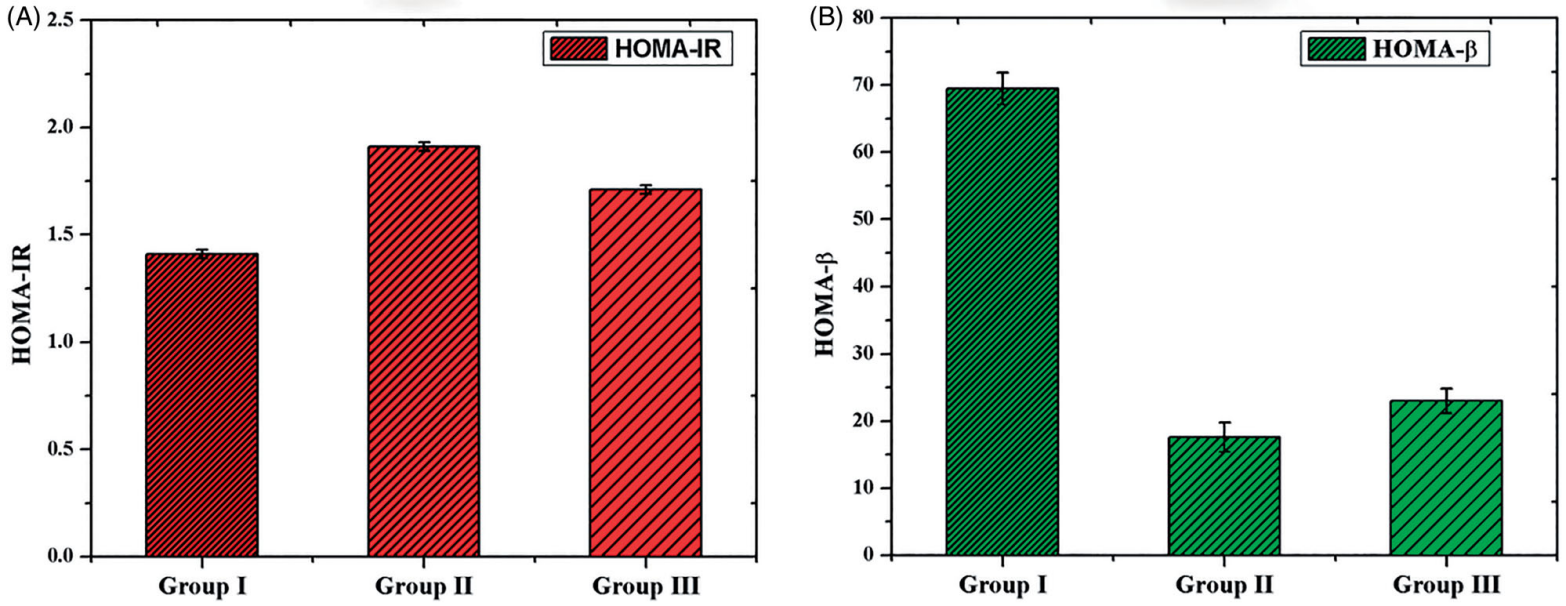
The homeostasis model valuation for insulin resistance (A) and HOMA for β-cell (B) in GDM and CS–ZnO–RS treated rats.

After the treatment of CS–ZnO–RS, body weight, blood glucose, and insulin were measured. The body weight was increased in all the groups of animals. However, there was no significant variance observed between the groups (Table 2). Simultaneously, the blood glucose level was also measured. The experimental group of rats (group II) displayed a significantly increased level of blood glucose than the control group. At the same time, we have noticed the decreased level of blood glucose level in GDM rats treated by CS–ZnO–RS (100 mg/kg bw) (Table 3). Alternatively, the level of insulin was decreased in Group II. However, the GDM rats treated with CS–ZnO–RS (100 mg/kg bw) showed an elevated level of insulin content than the untreated rats (Table 3). There is no significant variance of HOMA-IR observed in the control and experimental groups (Figure 6(A)). However, the GDM groups of rats showed a decreased level of HOMA-β than control (Figure 6(B)). In contrast, the GDM rats treated with CS–ZnO–RS showed an increased level of HOMA-β than untreated groups. Overall these findings concluded that CS–ZnO–RS reduced the symptoms of GDM. Similarly, Sha et al. reported that the treatment of Mangiferin ameliorates which effectively controls the blood glucose and insulin levels in gestational diabetes mice (Sha et al., 2019).

**Table 2.** Variation of body weight in control and experimental groups.

	Body weights	
	Before pregnancy	After pregnancy
Group-I	260.5 ± 0.8	380.7 ± 1.3
Group-II	270.6 ± 1.3	340.7 ± 1.6
Group-III	276.3 ± 0.75	378.2 ± 1.6

Data are expressed as mean ± standard deviation (SD).

**Table 3.** Determination of blood glucose and insulin level analysis in control and experimental groups.

	Blood glucose (mg/dL)	Insulin (ng/mL)
Group-I	99.9 ± 1.9	3.1 ± 0.2
Group-II	248.8 ± 2.2	2.6 ± 0.1
Group-III	119.5 ± 1.3	2.9 ± 0.1

Data are expressed as mean ± standard deviation (SD).

#### 3.5.2. Determination of lipid profile

**Figure 7.**
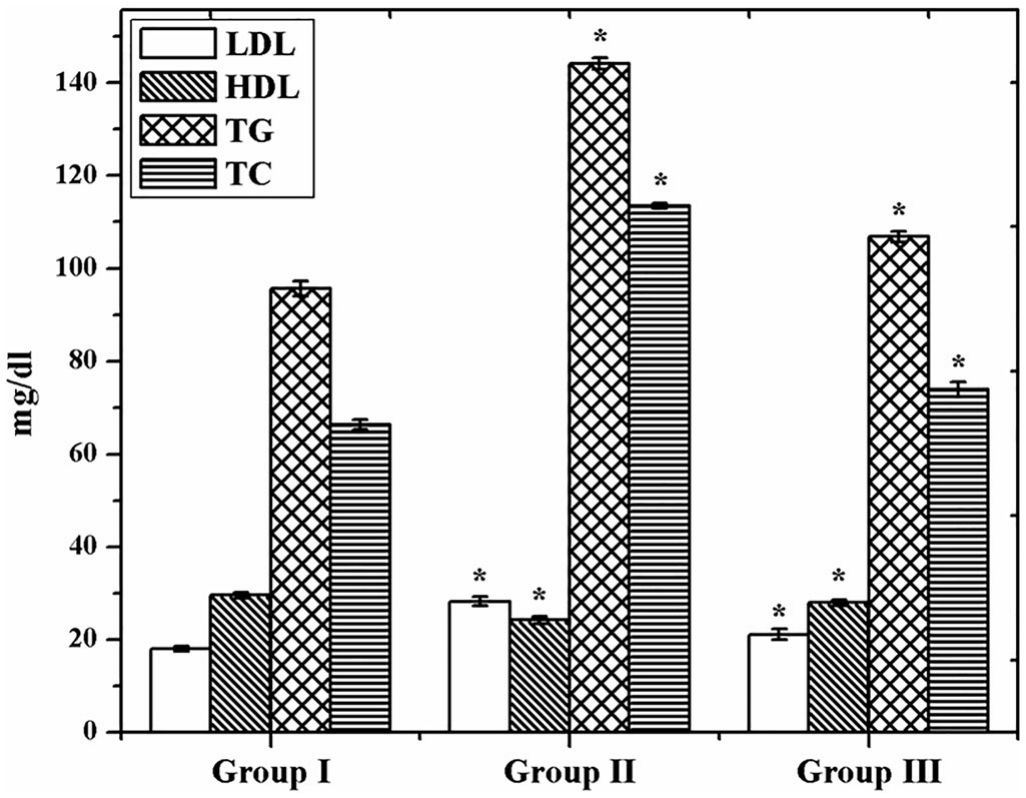
Evaluation of lipid profile in GDM and CS–ZnO–RS treated rats.

Through the examination of TC, TG, HDL, and LDL contents in control and experimental groups, we found significantly (*p<*.05) increased level of TC, TG, and LDL in GDM group than control (Figure 7). On the other hand, the HDL level was declined in the GDM group. However, the GDM rats were treated with CS–ZnO–RS which showed a decreased level of TC, TG, and LDL content. Simultaneously, the HDL level was significantly (*p<*.05) increased. Interestingly, the AI was reduced in group III. These results demonstrated that CS–ZnO–RS shows admirable effects on GDM, it can be applied for the possible therapeutic agent against GDM.

#### 3.5.3. Antioxidant evaluation

**Figure 8.**
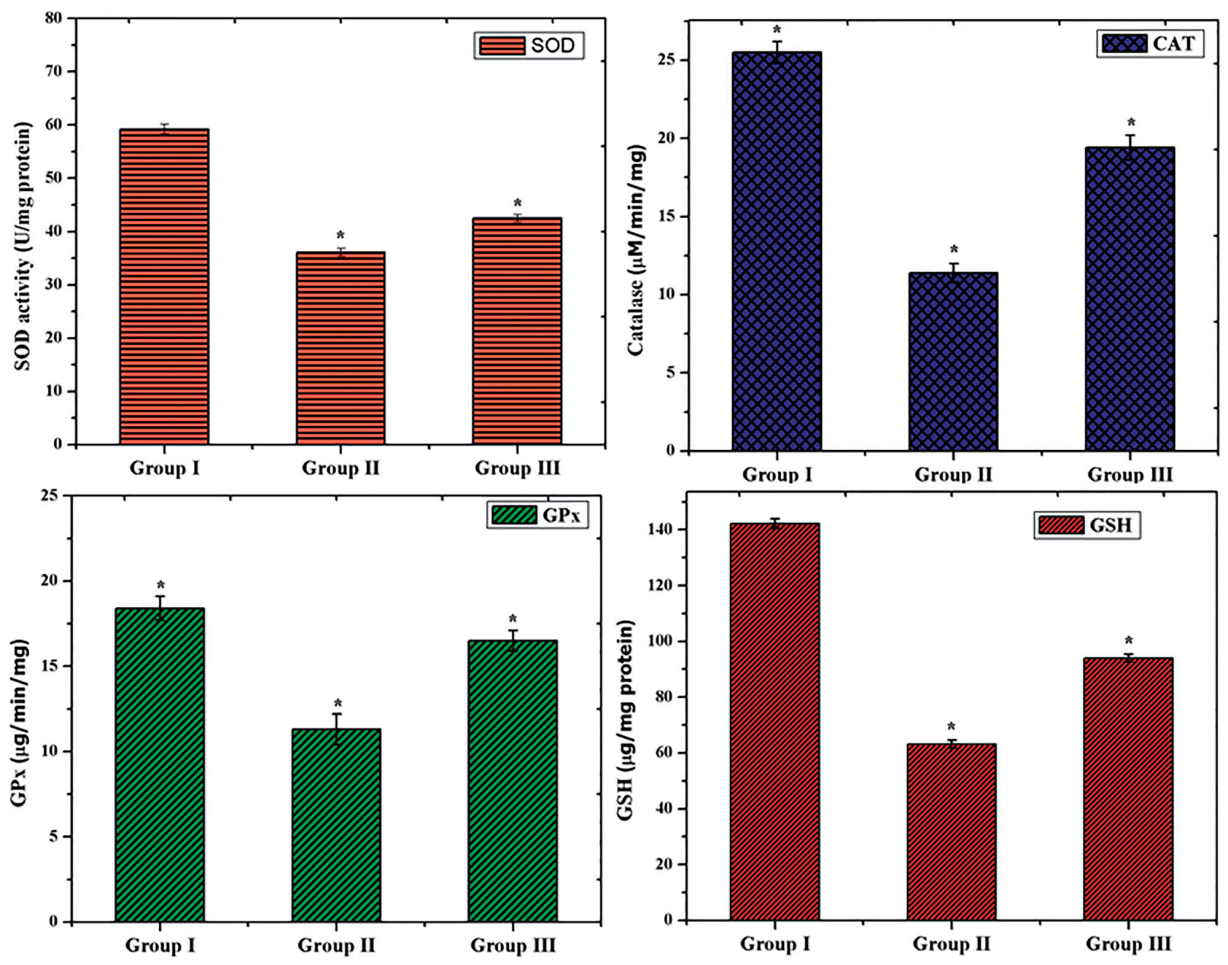
Assessment of antioxidant activities in in GDM and CS–ZnO–RS treated rats.

Overproduction of free radicals and lipid peroxidation (LPO) was induced by oxidative damage in the living organism (Rahal et al., 2014; Nimse & Pal, 2015; Simioni et al., 2018). The major enzymic antioxidants such as SOD, CAT, and GPx play an important role to overcome the oxidative stress (Nimse & Pal, 2015). As shown in Figure 8, the GDM rats (group II) exhibited a significantly (*p<.05*) decreased level of SOD, CAT, and GPx than control. However, the GDM challenged rats treated with CS–ZnO–RS (group III) showed a significantly increased level (*p<*.05) of SOD, CAT, and GPx activities. Reduced GSH is associated with GPx and GST which contributes to the major function in protecting the cells from the toxic chemicals (Searle & Willson, 1980). The decreased level of GSH and increased level of LPO, are primary features of diabetes mellitus (Mukherjee et al., 1994). The non-enzymic antioxidant (GSH) was also examined. After, streptozotocin exposure (group II), the rats displayed a decreased level of GSH than control (group I). Interestingly, the GDM rats were treated with CS–ZnO–RS significantly increased level of GSH than GDM group.

#### 3.5.4. RT-PCR analysis

**Figure 9.**
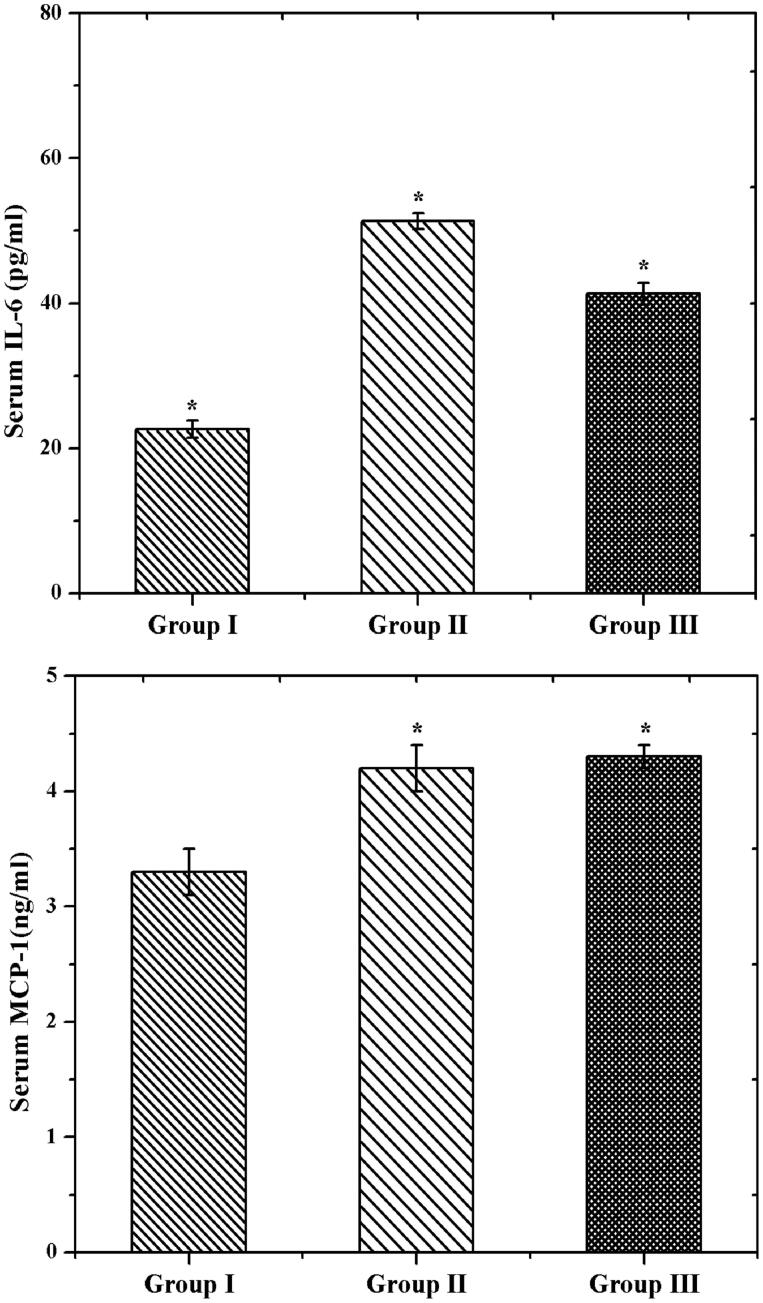
The mRNA expressions of IL-6 and MCP-1 in control and experimental rats using RT-PCR analysis.

Further, the inflammatory activity was investigated by RT-PCR analysis. The IL-6 and MCP-1 are the main contributors of GDM related disease (Wojcik et al., 2016). IL-6 promotes the acute-phase response and releases the CRP in pregnancy. Likewise, MCP-1 plays a principle role in the inflammatory function. As shown in Figure 9, we had a significantly elevated IL-6 and MCP-1 levels in group II (GDM rats). Similar levels were noticed in GDM (Klein et al., 2008). However, the GDM rats treated by CS–ZnO–RS exhibited a decreased level of IL-6 and MCP-1 (group III). These expressions confirmed that CS–ZnO–RS reduced the inflammatory factors in diabetes rats.

#### 3.5.5. Western blot analysis

**Figure 10.**
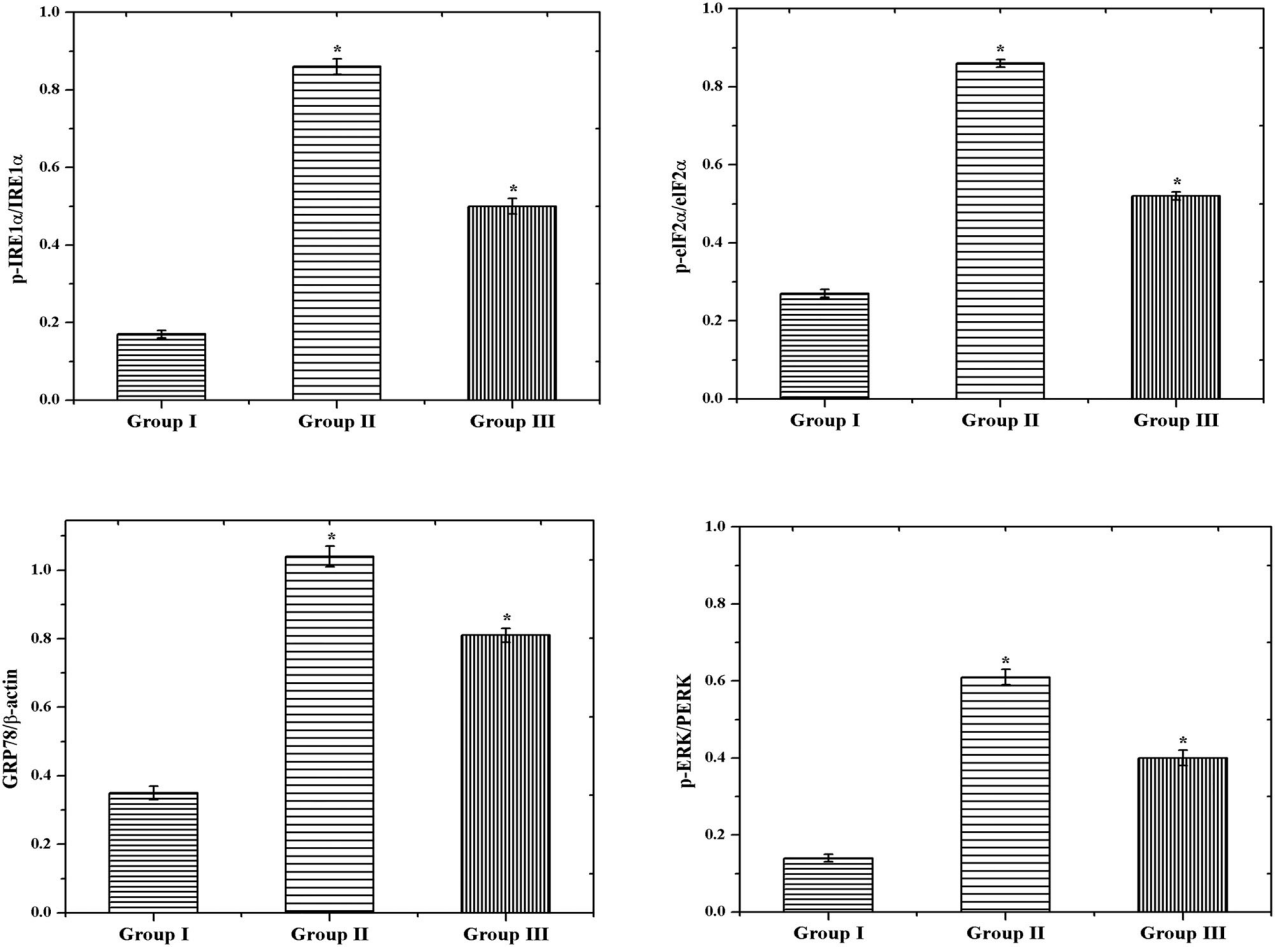
The protein expression of endoplasmic reticulum stress-related factors (GRP78, p-IRE1α, and p-eIF2α) by western blot method.

Generally, the endoplasmic reticulum (ER) stress was induced by both inflammation and oxidative stress. Furthermore, the protein synthesis and post-translational changes were regulated by ER. Even though the ER environment was distressed, the bioactivity of protein automatically affected which leads to accumulating the misfolded proteins (Lee, 2005). We examined the potential activity of CS–ZnO–RS on ER in normal and experiment rats by measuring the ER stress markers such as p-IRE1α, p-eIF2α, GRP78, and p-PERK. We had elevated levels of GRP78, p-IRE1α, p-eIF2α, and p-PERK in GDM challenged group of rats (group II). Later in the quantification of this protein, we had noticed the significant variance between GDM (group II) and control group (group I). However, the GDM group of rats treated by CS–ZnO–RS showed a decreased level of p-IRE1α, p-eIF2α, GRP78, and p-PERK (Figure 10).

#### 3.5.6. Histopathology analysis

**Figure 11.**
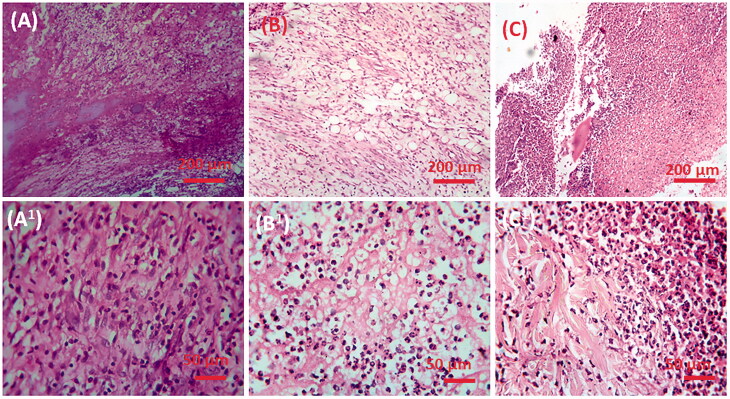
The histopathological examination of control and experimental rat liver; (A, A_1_) control, (B, B_1_) diabetic mellitus rats (C, C_1_) diabetic rats treated with CS–ZnO–RS in different microscopic magnifications (×40 and ×10).

Histopathological changes of liver in control, GDM, and CS–ZnO–RS treated groups are displayed in Figure 11. The control group of rats (group I) showed normal parenchyma cells with intact hepatocytes and sinusoids (Figure 11(A,A_1_)). By contrast, GDM induced rat showed certain pathological signs such as alteration in the cellular arrangement which around the central vein, vacuolation, vein walls were enlarged and condensed, capillaries and fibrosis formation in the immoral cells (Figure 11(B,B_1_)). However, the GDM rats treated with CS–ZnO–RS demonstrated that the cellular arrangement was rescued, diminished fibrosis and it too facilitated to carry the blood vessels to normal conditions (Figure 11(C,B_2_)).

## 4. Conclusions

In this study, the effect of resveratrol–zinc oxide complex encapsulated with chitosan (CS–ZnO–RS) nanoparticles on GDM was studied. CS–ZnO–RS showed an improvement of stability and effectiveness in GDM. The synthesized CS–ZnO–RS exhibited the average size at 38 nm. Meanwhile, the CS–ZnO–RS exposed 95% of drug-releasing activity within 24 h. Moreover, the CS–ZnO–RS reduced the diabetes signs in GDM challenged rats. The anti-diabetic activity was linked with the inflammatory factor IL-6 and MCP-1 and ER stress indicators such as GRP78, p-IRE1α, p-eIF2α, and p-PERK.
